# Supplementary material for: Impaired ABCA1/ABCG1-mediated lipid efflux in the mouse retinal pigment epithelium (RPE) leads to retinal degeneration
Source: eLife. 2019 Mar 13;8:e45100. doi: 10.7554/eLife.45100 (PMC6435327; doi:10.7554/eLife.45100)
Supplement: Supplementary file 1. — (A) Absolute concentrations of analyzed lipid classes from 2-months-old Ctr and RPEΔAbca1;Abcg1 mice. (B) RHO measurements from 2-months-old Ctr and RPEΔAbca1;Abcg1 neural retinas. (C) Primers used for genotyping. (D) Primers used for gene expression analysis. [file elife-45100-supp1.docx]

**Supplementary Information**

**Supplementary Tables**

**Supplementary file 1A. Absolute concentrations of analyzed lipid classes from 2-months-old Ctr and RPE^Δ^*^Abca1;Abcg1^* mice.** Shown are averages ± SD of N=4-10 mice.

| **Lipid class** | **Eyecup**  (pmol/µg of protein) | | **Neural retina**  (pmol/µg of protein) | | **Plasma**  (µM) | | |
| --- | --- | --- | --- | --- | --- | --- | --- |
|  | **Ctr** | **RPE^Δ^*^Abca1;Abcg1^*** | **Ctr** | **RPE^Δ^*^Abca1;Abcg1^*** | **Ctr** | **RPE^Δ^*^Abca1;Abcg1^*** | |
| *UC* | 31.1 ± 17.1 | 26.1 ± 4.0 | 20.3 ± 7.4 | 42.2 ± 33.3 | 14.2 ± 46.7 | 221.1 ± 190.7 |  |
| *CEs* | 43.9 ± 27.9 | 1239.9 ± 955.1 | 1.1 ± 0.3 | 2.7 ± 1.1 | 3196.9 ± 982.4 | 2915.8 ± 944.5 |  |
| *PLs (total)* | 408.1 ± 283.3 | 405.4 ± 710.3 | 178.3 ± 6.7 | 170.2 ± 13.5 | 844.1 ± 208.8 | 1089.3 ± 395.3 |  |
| *PC* | 55.9 ± 4.6 | 51.4 ± 6.22 | 90.0 ± 7.0 | 91.6 ± 3.0 | 924.6 ± 341.3 | 708.3 ± 163.3 |  |
| *PE* | 21.0 ± 2.3 | 20.2 ± 2.3 | 56.0 ± 4.5 | 59.3 ± 2.3 | 13.9 ± 6.6 | 10.0 ± 3.8 |  |
| *PS* | 1.9 ± 0.91 | 1.7 ± 1.5 | 7.6 ± 1.9 | 8.4 ± 1.1 | 0.2 ± 0.2 | 0.2 ± 0.2 |  |
| *PG* | 320.9 ± 714.6 | 329.8 ± 286.2 | 4.6 ± 2.8 | 5.6 ± 1.9 | 0.8 ± 0.2 | 0.8 ± 0.2 |  |
| *PI* | 5.6 ± 1.2 | 5.1 ± 1.5 | 12.1 ± 1.6 | 13.4 ± 0.8 | 149.7 ± 70.0 | 124.8 ± 46.7 |  |
| *Cer* | 2.2 ± 0.5 | 2.2 ± 0.7 | 0.4 ± 0.1 | 0.4 ± 0.1 | 3.4 ± 1.3 | 4.3 ± 2.2 |  |
| *SMs* | 14.1 ± 1.6 | 14.0 ± 2.2 | 5.2 ± 0.4 | 4.7 ± 0.7 | 126.4 ± 42.3 | 162.6 ± 64.6 |  |
| *DAGs* | 0.3 ± 0.1 | 0.3 ± 0.2 | 0.4 ± 0.1 | 0.4 ± 0.1 | 3.4 ± 1.6 | 5.3 ± 2.5 |  |
| *TGs* | 11.7 ± 6.0 | 25.2 ± 29.6 | 3.6 ± 0.2 | 3.4 ± 0.2 | 567.3 ± 300.7 | 1250.9 ± 915.7 |  |

**Supplementary file 1B. RHO measurements from 2-months-old Ctr and RPE^Δ^*^Abca1;Abcg1^* neural retinas.** Shown are averages ± SD of the indicated number of eyes.

| **Condition** | **Ctr** | | **RPE^Δ^*^Abca1;Abcg1^*** | |
| --- | --- | --- | --- | --- |
|  | **RHO (pmol/eye)** | **N eyes (mice)** | **RHO (pmol/eye)** | **N eyes (mice)** |
| *Dark* | 491.5 ± 120.1 | 6 (3) | 536.0 ± 58.5 | 6 (3) |
| *Bleached (0 min)* | 42.7 ± 28.8 | 4 (2) | 30.7 ± 18.8 | 6 (3) |
| *+ 30 min* | 190.1 ± 31.4 | 6 (3) | 133.5 ± 21.4 | 7 (4) |
| *+ 60 min* | 241.0 ± 35.9 | 6 (3) | 197.8 ± 34.3 | 7 (4) |
| *+ 120 min* | 352.1 ± 69.0 | 5 (3) | 313.9 ± 71.7 | 6 (3) |

**Supplementary file 1C. Primers used for genotyping.**

| **Gene** | **Primer sequence (5’ – 3’)** | **Products [bp]** | |
| --- | --- | --- | --- |
| *Abca1 flox* | for: GCCACTGCATCATAGTTGCT | wt: 376 | floxed: ~ 200 |
|  | rev: GTGGGGTGAGACATGTGGA |  |  |
| *Abca1 exc* | for: GAGGTTGCCCCTACGGATTTA | wt: 2086 | excised: 180 |
|  | rev: GTGGGGTGAGACATGTGGA |  |  |
| *Abcg1 flox* | for: TTTCCCAGAGATCCCTTTCA | wt: 600 | floxed: 700 |
|  | rev: GATCTAGGCAGAAGGCACTTG |  |  |
| *Abcg1 exc* | for: TGCCCCGTCCCCTTCTAA | wt: 1086 | excised: ~ 500 |
|  | rev: TGACCTTGGTACATTTTCCTGTC |  |  |
| *BEST1Cre* | for: ATGCCCAAGAAGAAGAGGAAGGTGTCC | wt: - | Cre: ~ 300 |
|  | rev: TGGCCCAAATGTTGCTGGATAGTTTTTA |  |  |

**Supplementary file 1D. Primers used for gene expression analysis.**

| **Gene** | **Primer sequence (5’ – 3’)** | **Product [bp]** |  |
| --- | --- | --- | --- |
| *Abca1* | for: gggagtcccagaaaaggaag | 179 | |
|  | rev: tgtggttggttcatccagaa |  | |
| *ABCA1* | for: GGTCATGGCTGAGGTGAACA | 159 |  |
|  | rev: TGGTCATTGTCCCTGCTGTC |  |  |
| *Abcg1* | for: ggtggaagaagaaaggatacaaga | 148 |  |
|  | rev: ttcatgccagtctccctgt |  |  |
| *Actb* | for: CAACGGCTCCGGCATGTGC | 153 |  |
|  | rev: CTCTTGCTCTGGGCCTCG |  |  |
| *ACTB* | for: CCTGGCACCCAGCACAAT | 144 |  |
|  | rev: GGGCCGGACTCGTCATAC |  |  |
| *Casp1* | for: GGCAGGAATTCTGGAGCTTCAA | 138 |  |
|  | rev: GTCAGTCCTGGAAATGTGCC |  |  |
| *Cre* | for: taaactggtcgagcgatgga | 187 |  |
|  | rev: accagagtcatccttagcgc |  |  |
| *Gfap* | for: CCACCAAACTGGCTGATGTCTAC | 240 |  |
|  | rev: TTCTCTCCAAATCCACACGAGC |  |  |
| *Il1b* | for: ACTACAGGCTCCGAGATGA | 141 |  |
|  | rev: CGTTGCTTGGTTCTCCTTG |  |  |
| *Mct3* | for: TCCAGAGTCAGGTCCAGGTT | 75 |  |
|  | rev: GAAGACGCTCACAGCCTTAG |  |  |
| *Rpe65* | for: aaagcagacaaggaagatccaa | 178 |  |
|  | rev: cccaaagactccacgaagaa |  |  |
| *RPL28* | for: GCAATTCCTTCCGCTACAAC | 198 |  |
|  | rev: TGTTCTTGCGGATCATGTGT |  |  |
